# Ocular Biometry and Genomic Association in Primary Angle Closure Disease. A Descriptive study

**DOI:** 10.22336/rjo.2024.75

**Published:** 2024

**Authors:** Sangaraju Suneel, Subashini Kaliaperumal, Sunitha Kodidela, Alladi Charanraj Goud, Mary Stephen

**Affiliations:** 1 Department of Ophthalmology, Jawaharlal Institute of Postgraduate Medical Education and Research (JIPMER), Puducherry, India; 2 Department of Pharmacology, Jawaharlal Institute of Postgraduate Medical Education and Research (JIPMER), Puducherry, India

**Keywords:** glaucoma, angle-closure disease, genotyping, polymorphism, biometry, POAG = Primary Open Angle Glaucoma, PACG = Primary Angle Closure Glaucoma, PACS = Primary Angle Closure Suspect, PAC = Primary Angle Closure, PACD = Primary Angle Closure Disease, AXL = Axial Length, ACD = Anterior Chamber Depth, MMP 9 = Matrix metalloproteinase-9, HGF = Hepatocyte growth factor, eNOS = Endothelial nitric oxidase synthase, MTHFR = Methyl-tetrahydrofolate reductase, GWAS = Genome-wide association study, LT = Lens thickness, CCT = Central corneal thickness, Mean K = Mean keratometry, RLP = Relative lens position, LAF = Lens axial length factor

## Abstract

**Purpose:**

To study the ocular biometric parameters in PACD patients and to compare them with normal control subjects. To identify the role of genetic polymorphisms in PCMTD1 and COL11A1 genes in our population in PACD pathogenesis.

**Materials and methods:**

This cross-sectional comparative study included patients with PACD age-matched normal subjects. Patients who underwent prior laser iridotomy or intraocular surgery and those using miotics were excluded from the study. The comprehensive ophthalmological evaluation included slit lamp biomicroscopy, applanation tonometry, gonioscopy, and optic disc evaluation. PACD patients were classified as per the International Society for Geographical and Epidemiological Ophthalmology (ISGEO) classification into PACS, PAC, and PACG. Ocular biometry was performed for parameters like AXL, ACD, lens thickness (LT), central corneal thickness (CCT), and mean keratometry (K) values using the Partial Coherence Interferometry biometer. Variables like relative lens position (RLP) and lens axial length factor (LAF) were calculated from the above parameters. Genotyping was done for PACD patients and control subjects to look for PCMTD1, NM_001286783.1:c.215G>A and COL11A1, NM_080629.2:c.2386C>G genetic polymorphism.

**Results:**

A total of 200 eyes of 100 PACD patients and the same number of control subjects were included in the study. PACD patients had significantly shorter AXL (21.68 mm vs. 23.25 ± 0.63 in controls, p < 0.05), short anterior chamber depth (2.33 ± 0.5 mm vs. 2.97 ± 0.92, p < 0.05), and increased Lens thickness (4.45 ± 0.41 mm vs. 4.11 ± 0.45 mm, p = 0.00). The Relative lens position (2.10 ± 0.2 vs. 2.16 ± 0.17, p < 0.03), Lens axial length factor (2.06 ± 0.21 vs. 1.76 ± 0.2, p = 0.00), Mean keratometry (D) (45.73 ± 1.64 vs. 44.33 ± 1.37, p = 0.00) were significantly different. Central corneal thickness (µ) (516.15 ± 34 vs. 511.9 ± 35.28, p = 0.8) was insignificant. Among the subgroups of PACD, PACG patients had steeper corneas when compared to PACS and PAC (46.67 ± 2.45 vs. 45.55 ± 1.54 vs. 45.64 ± 1.1, p < 0.01), while other parameters were not significant. Genotyping of PCMTD1, NM_001286783.1:c.215C>T, and COL11A1, NM_080629.2:c.2386C>G polymorphism yielded no significant association with PACD.

**Discussion:**

Though association studies have identified several candidate genes, these are either not extended to other populations or controversial. In our research, ocular biometric parameters vary in primary angle closure disease patients, with corneal curvature being the only exemption in the studied variable without significant differences. We also found that the allelic frequency for PCMTD1, NM_001286783.1:c.215C>T was 95.75% and 4.25% for C and T, respectively, which is different from available data (97% and 3% for C and T in South Asians). For COL11A1, NM_080629.2:c.2386C>G, we found a % allelic frequency of 96% and 4% for C and G, respectively. The study subjects with heterozygous genotypes of PCMTD1 and COL11A1 showed an Odds Ratio of 1.48 (95% CI: 0.53-4.04), p=0.46 and 1.31 (95% CI: 0.47-3.68), p=0.8 when compared to controls, which were not significant.

**Conclusion:**

Ocular biometric parameters vary significantly in PACD patients compared to normal subjects. Except for corneal curvature, there was no significant difference among PACS, PAC, and PACG.

## Introduction

Glaucoma is the third leading cause of blindness worldwide and the leading cause of irreversible blindness [1,2]. Based on the anatomy of the anterior chamber angle, two main forms of primary glaucoma exist primary open-angle glaucoma (POAG) and primary angle closure glaucoma (PACG). The prevalence of primary angle closure glaucoma (PACG) is higher in Asians when compared to Europeans and Africans, with over 80% of PACG patients worldwide living in Asia [3]. PACG is considered a significant health problem as the frequency of blindness in PACG is more than in POAG [3]. Pathogenesis of primary angle closure disease is multifactorial. It is considered a disorder of ocular anatomy, characterized by iris appositional or synechial closure of drainage angle leading to a decrease in aqueous drainage and thereby increasing intra-ocular pressure. Eyes with primary angle closure inherently by nature have a short axial length (AXL) [4], steeper corneas [5], shallow anterior chamber depth (ACD) [6,7], thicker crystalline lens [4,6], and anteriorly positioned crystalline lens. Although the above parameters are implicated in the pathogenesis of angle-closure disease, their characteristics in the subtypes of primary angle-closure disease (PACD) have yet to be thoroughly studied. Ocular biometry has been extensively studied, and their comparison between PACG and controls has been extensively reported. However, all the previous studies have followed various classification systems in defining PACD subtypes, leading to a need for uniformity. As far as we know, there needs to be more data on biometric comparison among subgroups of PACD.

Furthermore, it is suggested that PACD is a complex disease with genetic and environmental factors having a role in the pathogenesis, most likely multiple anatomical and physiological characteristics [8]. It has long been suspected that there is a significant genetic risk in the pathogenesis of PACD because of its association with race, gender, and family history [9]. Literature shows that various genes such as hepatocyte growth factor (HGF) [10], matrix metalloproteinase-9 (MMP-9) [11], endothelial nitric oxidase synthase (eNOS) [12], and methyl-tetrahydrofolate reductase (MTHFR) [13] have been associated with PACG. However, these findings must be more consistent in the literature and have not been consistently replicated by independent studies. Two-stage, genome-wide association study (GWAS) conducted on a large cohort of 3771 PACG cases and 18551 controls from Hong Kong, India, Malaysia, Singapore, and Vietnam has shown significant association between PACG and three genetic markers (rs11024102 in PLEKHA7, rs3753841 in COL11A1, and rs1015213 located between PCMTD1 and ST18) [8].

We conducted this study to assess ocular biometric parameters in PACD subjects using the ISGEO classification system for disease sub-grouping. Additionally, we explored the potential role of two genetic markers in the pathogenesis of PACD by performing genotyping on two single nucleotide polymorphisms (SNPs): PCMTD1 (NM_001286783.1.215C>T) and COL11A1 (NM_080629.2.2386C>G) as part of a pilot investigation.

## Materials and methods

Ethical clearance was obtained from the Institute Ethical Committee, and its implementation strictly complied with the standards of the Declaration of Helsinki. This cross-sectional comparative study was done on a South Indian population, and consecutive patients with PACD attending the glaucoma clinic of a tertiary care hospital were recruited from 2016-2018. Subjects included were patients with PACD, which formed the PACD group, and age-matched normal subjects without primary or secondary angle closure selected from the general OPD, which formed the Control group. Patients who underwent prior laser iridotomy or intraocular surgery and those using miotics were excluded from the study. The comprehensive ophthalmological evaluation included slit lamp biomicroscopy, applanation tonometry, gonioscopy, and optic disc evaluation.

PACD patients were classified as per the International Society for Geographical and Epidemiological Ophthalmology (ISGEO) classification into PACS, PAC, and PACG [14].

1. **Primary angle closure suspect (PACS) -** An eye in which appositional contact between the peripheral iris and posterior trabecular meshwork is considered possible {defined as an angle in which >270° of the posterior trabecular meshwork (the part which is often pigmented) cannot be seen}.
2. **Primary angle closure (PAC) -** An eye with an occlusal drainage angle and features indicating that trabecular obstruction by the peripheral iris has occurred, such as peripheral anterior synechiae, elevated intraocular pressure, iris whorling (distortion of the radially orientated iris fibers), “glaucomflecken” lens opacities, or excessive pigment deposition on the trabecular surface. The optic disc does not have glaucomatous damage.
3. **Primary angle closure glaucoma (PACG) -** PAC and evidence of glaucoma, as defined above.

### Ocular biometry

Ocular biometry was performed on all eyes of PACD patients and control subjects. Biometric parameters like AXL, ACD, lens thickness (LT), central corneal thickness (CCT), and mean keratometry (K) values were obtained for both eyes using the Partial Coherence Interferometry biometer (LENSTAR LS 900 optical biometer, Haag-Streit USA, Mason, Ohio). Variables like relative lens position (RLP) and lens axial length factor (LAF) were calculated and derived from the abovementioned parameters.

### Genotyping

Genotyping was done for PACD patients and control subjects. DNA was extracted from cellular fraction from five ml of peripheral venous blood samples. Quantification of DNA and quality checking of the samples by Multianalyzer were performed. The genotyping of selected SNPs (PCMTD1, NM_001286783.1: c.215C>T and COL11A1, NM_080629.2:c.2386C> G.) was carried out using validated TaqMan allele discrimination assays according to the manufacturer’s instructions (Applied Biosystems; Foster City, CA, USA) on a real-time PCR instrument (ABI Prism 7300; Foster City, CA, USA).

### Sample size calculation

The sample size was calculated to be 100 in each group (200 subjects and 400 eyes). It was computed assuming α = 0.05, a power of 80%, and the lowest mean difference value of 0.06 from the ocular characteristic (lens/axial length factor) studied in a similar study by Chen et al. [2]. The standard deviations of the two groups were 0.14 and 0.16. The sample size was calculated using OpenEpi, Ver3.03a.

### Statistical analysis

Categorical data, such as gender and genotype associations, were analyzed using the Chi-square association test. The unpaired “t” test was used to compare variables like Age, AXL, ACD, LT, RLP, LAF, K, and CCT in the two groups. Subgroup analysis among PACS, PAC, and PACG was done using One-way ANOVA (Analysis of variance). All statistical operations were performed using SPSS version 19.0 (SPSS Inc., Chicago, IL, USA).

## Results

200 eyes of 100 PACD patients were included. Of these, 65 patients (65%) were diagnosed with PACS, 21 patients (21%) had PAC, and 14 (14%) had PACG. The Control group comprised 200 eyes of 100 randomly selected age-matched normal subjects. The mean age among subjects with PACD was 58.22 ± 10.48 years and was comparable with that of control subjects (59.35 ± 10.9 years) with no statistically significant difference (p= 0.46). Also, subgroups of PACD subjects were comparable in terms of age distribution. The study group had 62 (62%) females and 38 (38%) males, whereas the control group had 43 (43%) females and 57 (57%) males.

### Biometric characteristics

Patients with PACD had significantly shorter AXL, shallow ACDs, thicker lenses, steeper corneas, and anteriorly positioned crystalline lens (RLP) compared to normal control subjects with all the p values < 0.001. Central corneal thickness was comparable between the groups. Biometric parameters in PACD patients and control subjects are shown in **Table 1**. A comparison of the mean biometric parameters amongst subgroups in PACD is shown in **Table 2**. In the PACD group, patients with PACG had significantly steeper corneas when compared to PACS and PAC with p values < 0.05. However, subgroup analysis of other biometric parameters yielded insignificant differences among PACS, PAC, and PACG patients (**Table 3**).

**Table 1:** Ocular biometric parameters in PACD and control group

	PACD GROUP	CONTROL GROUP	P value
**Axial length (mm)**	21.68 ± 0.85	23.25 ± 0.63	**0.00**
**Anterior chamber depth (mm)**	2.33 ± 0.51	2.97 ± 0.92	**0.00**
**Lens thickness (mm)**	4.45 ± 0.41	4.11 ± 0.45	**0.00**
**Relative lens position**	2.10 ± 0.2	2.16 ± 0.17	**0.03**
**Lens axial length factor**	2.06 ± 0.21	1.76 ± 0.2	**0.00**
**Mean keratometry (D)**	45.73 ± 1.64	44.33 ± 1.37	**0.00**
**Central corneal thickness (µ)**	516.15 ± 34	511.9 ± 35.28	0.8

**Table 2:** Comparison of ocular biometric parameters among sub-groups

	CONTROL	PACS	PAC	PACG
**AXL**	23.25 ± 0.63	21.72 ± 0.88	21.66 ± 0.67	21.59 ± 0.96
**ACD**	2.97 ± 0.92	2.37 ± 0.49	2.25 ± 0.55	2.28 ± 0.56
**LT**	4.11 ± 0.45	4.41 ± 0.41	4.5 ± 0.36	4.6 ± 0.42
**RLP**	2.16 ± 0.17	2.11 ± 0.19	2.08 ± 0.2	2.12 ± 0.23
**LAF**	1.76 ± 0.2	2.03 ± 0.2	2.08 ± 0.2	2.13 ± 0.23
**K**	44.33 ± 1.37	45.55 ± 1.54	45.64 ± 1.1	46.67 ± 2.45

**Table 3:** Significance of subgroup analysis for biometric parameters

	PACS vs. PAC	PACS vs. PACG	PAC vs. PACG
**AXL**	0.65	0.43	0.56
**ACD**	0.13	0.49	0.59
**LT**	0.12	0.11	0.42
**RLP**	0.3	0.43	0.36
**LAF**	0.11	0.6	0.35
**K**	0.97	**0.01**	**0.02**

### Genotyping

Genotyping of PCMTD1, NM_001286783.1:c.215C>T, and COL11A1, NM_080629.2:c.2386C>G polymorphism yielded no significant association with PACD. However, allelic frequencies for PCMTD1, NM_001286783.1:c.215C>T were 95.75% and 4.25% for C and T, and for COL11A1, NM_080629.2:c.2386C>G were 96% and 4% for C and G respectively in our study population. The association between genotypes and odds of developing PACD are shown in **Table 4**.

**Table 4:** The association between genotypes and PACD

*PCMTD* rs79820636 (C>T)					
**Population**	**Number(N)**	**CC**	**CT**	**Odds ratio (95 %CI)**	**p-value**
Controls	100	93	7	1.48 (0.53-4.04)	0.6
Cases	100	90	10		
***COL11A1* rs387906611(C>G)**					
**Population**	**Number (N)**	**CC**	**CG**	**Odds ratio (95% CI)**	**p-value**
Controls	100	93	7	1.31 (0.47-3.68)	0.8
Cases	100	91	9		

## Discussion

Worldwide, about 0.7% of the population aged above 40 years are estimated to have angle closure disease, which accounts for 20.2 million people [1]. Significant angle closure glaucoma is more common in India than in Western countries [15,16]. According to the literature, demographic factors such as older age, female gender, and a family history of angle closure, along with specific ocular characteristics like hyperopia, shorter axial length (AXL), shallow central and peripheral anterior chamber depths, steeper corneal curvature, a thicker crystalline lens, and ciliary body configurations typical of plateau iris, have been associated with the etiopathogenesis of PACD [2-5]. Our study’s findings support the above ocular biometric findings. Among the PACD patients, analysis of ocular biometric parameters in PACS, PAC, and PACG showed significant differences in central corneal thickness.

In our study, the mean AXL in the PACD group was 21.68 mm, which is 1.57 mm less than the control group (23.25 mm). A previous study from the South Indian population by George et al. reported similar results [4]. However, the mean AXL among the subgroups of PACD was comparable, with no significant difference, which is consistent with the available literature. The mean ACD in PACD patients in our study was 2.33 mm, which is significantly shallower compared to 2.97 mm in the control group. This finding, too, is per the study on the South Indian population by George et al. [4]. The ACD difference among the subgroups of PACD was insignificant. A study by He et al. on the Chinese population has reported that ACD tends to be shallower in populations with higher rates of angle closure and highlights the role of ACD estimation as a screening tool in identifying persons at risk of angle closure disease [1,7]. Increased Lens thickness (LT) and Lens Axial length Factor (LAF) with low Relative lens position (RLP) have been implicated as risk factors for the development of angle closure disease. Mean LT and LAF in PACD patients in our study were 4.45 mm and 2.06, respectively, significantly more than the control group (4.11 mm and 1.76). These findings were consistent with the previously reported studies by various others [2,4,5]. Subgroup analysis of these lens-related parameters, however, yielded insignificant differences. However, Chen et al., in their study, reported significant differences in the LAF between PAC and PACG subgroups [2]. The lack of substantial differences in LAF values among subgroups can be attributed to our study’s small sample size. The mean K value in the study group was 1.39 D higher than the control group; this difference was statistically significant, with p values less than 0.05. Subgroup analysis yielded substantial differences in mean K values between PACS and PACG subgroups and between PAC and PACG subgroups with p values less than 0.05. There was no significant difference in mean K values between PACS and PAC subgroups. This observation of steeper corneas in PACD compared to normal subjects is consistent with existing literature. However, a comparison of K values among subgroups, PACS, PAC, and PACG, was not done previously.

More research is needed into the genetic basis of PACD. In a South Indian population, Duvesh et al. used single nucleotide polymorphisms (SNPs) rs11024102 in PLEKHA7, rs3753841 in COL11A1, and rs1015213 between the PCMTD1 and ST18 genes, which were shown to be associated with PACG. They reported a significant genetic association with rs1015213 and an insignificant association with rs11024102 and rs3753841.

### Strengths

The LENSTAR LS 900 optical biometer, which provided higher resolution and more excellent repeatability compared to older methods like ultrasonography, enhanced the accuracy and reliability of the ocular parameters measured in our study.

Another strength is the adoption of the ISGEO Classification System for subgrouping PACD (Primary Angle-Closure Disease) patients. This standardized classification system helped avoid confusion and inconsistencies in previous studies and enhanced the clarity and comparability of our findings.

Including genotyping for two new SNPs (Single Nucleotide Polymorphisms) in the PCMTD1 and COL11A1 genes is a valuable contribution to the field. This can lead to new insights into the genetic factors associated with the disease and may provide a basis for further research.

### Limitations

A significant limitation of the study was the unequal distribution of cases among subgroups, which can introduce bias and affect the reliability of subgroups. When subgroups have significantly different sample sizes, it can be challenging to identify statistically significant differences between them. Secondly, the sample size for genetic analysis needed to be improved, making it difficult to draw meaningful conclusions about genetic factors.

## Conclusion

In conclusion, ocular biometric parameters show significant variation in PACD patients compared to normal subjects. However, aside from corneal curvature, there were no significant differences among PACS, PAC, and PACG groups.

Future studies should aim to recruit a larger and more evenly distributed sample across PACS, PAC, and PACG groups. This will enhance the statistical power and reliability of the findings, allowing for more definitive conclusions about the differences in ocular biometric parameters among these subgroups. Given the promising initial results from genotyping the PCMTD1 and COL11A1 SNPs, future research can focus on expanding the genetic analysis with a larger cohort. This would provide a more robust understanding of the genetic factors contributing to PACD and may help identify new genetic markers associated with the disease.
